# Characterization and Bioactivity of Polysaccharides Separated through a (Sequential) Biorefinery Process from *Fucus spiralis* Brown Macroalgae

**DOI:** 10.3390/polym14194106

**Published:** 2022-09-30

**Authors:** Cătălina Filote, Elhafnaoui Lanez, Valentin I. Popa, Touhami Lanez, Irina Volf

**Affiliations:** 1 Department of Environmental Engineering and Management, Faculty of Chemical Engineering and Environmental Protection, “Gheorghe Asachi” Technical University of Iasi, 73 Prof. D. Mangeron Bldv., 700050 Iasi, Romania; 2 VTRS Laboratory, Faculty of Sciences, University of El Oued, B.P. 789, El Oued 39000, Algeria

**Keywords:** brown algae, fucoidan, alginate, antioxidants, DNA binding study, bioactivity

## Abstract

Marine macroalgae biomass is a valuable renewable resource that can be used for the development of bioeconomy through the valorisation of valuable compounds. The aim of the current study is separate macroalgal polysaccharides with bioactive properties from brown macroalgae *Fucus spiralis* based on a designed biocascading biorefinery approach. Thus, we applied an integrated processing method for the separation of fucoidan and alginate, in addition to characterization through IR spectroscopy and ^1^H NMR. The bioactivity potential (antioxidant activity using superoxide anion and DPPH radical scavenging analysis) of the two polysaccharides was evaluated, together with DNA binding studies performed though voltametric techniques and electronic spectroscopy titration. In terms of results, functional groups S=O (1226 cm^−1^), N=S=O (1136 cm^−1^) and C-O-SO_3_ (1024 cm^−1^), which are characteristic of fucoidan, were identified in the first polysaccharidic extract, whereas guluronic units (G) (1017 cm^−1^) and mannuronic units (M) (872 and 812 cm^−1^) confirmed the separation of alginate. The DNA binding studies of the isolated polysaccharides revealed an electrostatic and an intercalation interaction of DNA with fucoidan and alginate, respectively. Both antioxidant activity assays revealed improved antioxidant activity for both fucoidan and alginate compared to the standard α-tocopherol.

## 1. Introduction

Marine macroalgae, also known as “seaweeds”, represent a promising biomass that can address future trends and industry demand, as well as various sustainability issues. Marine macroalgae have been recognized as an alternative for the production of renewable energy, bio-based products and bioactive molecules, owing to their remarkable regeneration properties and high photosynthetic efficiency, as well as the lack of land requirements for growth [1,2,3,4]. Yields in terms of growth potential of seaweeds are around 20 t per hectare per year [5]. Furthermore, the valorisation of invasive marine macroalgae could provide additional advantages through processing [6], whereas others are suitable for cocultivation with fish species in an integrated multitrophic aquaculture system (IMTA), where they play a bioremediation role [7]. The chemical composition of the marine macroalgae, including the bioactivity of valuable compounds, depends on the harvesting season, the algae species and the environmental conditions [8]. Furthermore, the separation method has a significant influence on the final chemical structure of the targeted compounds [9].

Processing of marine macroalgae through a biorefinery approach could enable the generation of cost-effective products and a sustainable valorisation with minimal waste or even the fulfilment of the zero-waste concept [10,11,12,13]. The amount of easily degradable carbohydrates in macroalgae typically ranges between 25 and 60% dry wt [5]. The lack of or minimal amount of lignin in macroalgae facilitates separation of carbohydrates compared with terrestrial biomass [14]. Therefore, their chemical composition makes marine macroalgae suitable for generation of fermentation products, such as bioethanol, methane, biohydrogen, bio-oil and biodiesel [5], as well as valuable bioactive molecules.

Brown macroalgae have been traditionally used as food and medicine in Asian countries. Globally, they are mainly applied as feed and crop fertilizer, owing to their high ash and mineral content [14,15]. Additionally, this biomass is highly complex in its chemical structure and includes valuable bioactive metabolites, some of which are unique in the natural world. Polysaccharides represent the main constituent of the cell wall of marine macroalgae and have important bioactive properties. Fucoidan, as well as some polyphenols, from brown algae can be used for the production of pharmaceuticals, nutraceuticals and cosmetics, owing to their high bioactivity potential [16,17,18]. Alginate, a macroalgal hydrocolloid, is mainly used in food, pharmaceutical and medical applications, as well as in textile and paper production [19,20,21]. Furthermore, its gelling and thickening properties have been increasingly exploited to generate innovative biofilms and hydrogels [22,23]. The separation of bioactive compounds is recommended to be performed in the initial steps of the biorefining process in order to ensure the feasibility of the process [24,25].

*Fucus spiralis*, which belongs to the order *Fucales*, is an edible and perennial brown macroalga common throughout the temperate regions of the northern hemisphere [26,27] occupying the littoral to sublittoral areas of rocky coasts [28]. Its chemical composition includes proteins with antioxidant properties and angiotensin-converting enzyme (ACE) inhibitory activity [29]; polyphenols, such as phlorotannins with antioxidant activity [30,31,32]; and anti-inflammatory properties [33]; as well as polysaccharides with anticoagulant [34], anticancer [35,36] and antioxidant activity [37].

Sequential processes applied to date for biorefining of brown algae biomass have consisted of the coproduction of bioactive compounds and/or biofuel, mostly from species of the genera *Laminaria* [15,21,38,39,40,41], *Sargassum* [13,42,43], *Macrocystis* [44,45], *Ascophyllum* [15] and *Ecklonia* [44,46].

The purpose of this work is (i) to design and perform an integrated valorisation of *Fucus spiralis* biomass, applying soft or green processes for the separation of high-value molecules; and for the targeted compounds, (ii) the evaluation of biological potential through DNA binding studies (an electrochemical DNA interaction study and an electronic spectroscopy DNA interaction assay) and antioxidant activity studies (an superoxide anion radical interaction study and a DPPH assay). To the best of our knowledge, no studies have been conducted on a biorefinery approach for *F. spiralis* macroalgae, and no evidence of the bioactive properties of the targeted molecules have been reported to date.

Therefore, in the current study, we highlight a pathway that can be applied in order to process brown macroalgae *Fucus spiralis* through a biorefinery approach, in addition to reporting the bioactive properties of the obtained biocompounds.

## 2. Materials and Methods

*Fucus spiralis* algae were collected during the winter season in the Viana do Castelo area, on the northern coast of Portugal. The biomass was washed with tap water, followed by a few washes with distilled water in order to eliminate impurities, sand and epiphytes. Algal biomass was further prepared by drying in an oven at 50 °C for 24 h. The dried algae were ground into particles with a size ≤ 0.5 cm and kept in a desiccator in sealed bags.

All reagents and solvents were of analytical grade and were used without further purification. Dimethyl sulfoxide (DMSO) and ethanol (HPLC-grade; Sigma-Aldrich, St. Louis, MO, USA) were used as solvents in voltametric and spectroscopic assays, whereas tetrabutylammonium tetra-fluoroborate (Bu_4_NBF_4_) (electrochemical grade 99%; Sigma-Aldrich) was used as a supporting electrolyte. Research-grade nitrogen and oxygen gases (99.99%) were provided by Linde Gas Algérie.

### 2.1. Methods of Extraction

#### 2.1.1. Extracts Containing Polyphenols

Polyphenols were extracted with 70% ethanol as solvent in a batch sonication system (Elmasonic 120 (H), Lauda Konigshofen, Germany; power, 80–320 W; frequency, 35 kHz), using a solid to liquid ratio of 1:10 at 40 °C, with an extraction time of 45 min [47]. The crude extract was filtered, and the residual biomass was dried at 40 °C and directly used for further extraction steps. The final filtered extract containing polyphenols is hereafter abbreviated as E_1_.

#### 2.1.2. Extracts Containing Polysaccharides

The two main macroalgal polysaccharides, fucoidan and alginate, were sequentially extracted from the obtained residue after the separation of polyphenols by adapting a protocol reported by Yuan and Macquarrie [48]. First, fucoidan was extracted using HCl 0.1 M as solvent, with a solid/liquid ratio of 1:10, at 40 °C, with a 20 min extraction time.

The alginate was isolated using a cost-effective and soft separation method with 4% Na_2_CO_3_ solution. Both extracts were filtered and precipitated with a 1:1 *v/v* ethanol:extract ratio. The residual biomass and the filtered extracts were washed with distilled water and dried at 40 °C. The final extracts are hereafter abbreviated as E_2_ (fucoidan) and E_3_ (alginate).

### 2.2. Chemical Characterization of Extracts

In order to determine the total polyphenolic content (TPC), E_1_ analysed according to the Folin–Ciocalteu method and quantified in mg gallic acid equivalents per gram of biomass (mg GAE g^−1^) [49].

We evaluated the total reducing sugar content in the supernatant produced after the separation and filtration of the E_2_ and E_3_ extracts, as well as in the wastewater resulting from the purification processes. The concentration of the reducing sugars was determined following the method described by the IUPAC [50], using an Avanta UV-VIS GBS spectrophotometer at 540 nm.

Elemental analysis was carried out using the Pregl method [51] for carbon and hydrogen, the Kjeldahl method [52] for nitrogen and the Schöniger method [53,54] for sulphur, with all results expressed in percentage.

The FTIR spectra were obtained in duplicate using an IRAffinity-1 Shimadzu FTIR instrument in the wavenumber range of 400–4000 cm^−1^.

Proton nuclear magnetic resonance (^1^H NMR) spectra were recorded by a Bruker Avance DRX 400 MHz spectrometer using deuterated water as solvent and tetramethylsilane as internal reference. Chemical shifts are reported in parts per million (ppm) relative to the residual peak of the deuterated water used as solvent. For the preparation of samples, 5 mg each of dry extracts E_2_ and E_3_ was dissolved in 800 μL of D_2_O.

### 2.3. Determination of Extract Bioactivity

#### 2.3.1. DNA Binding Studies

Double-stranded DNA (ds. DNA) was extracted from chicken blood using a nuclear cell lysis method [55]. Absorption spectroscopy and the molar absorption coefficient value of 6600 M^−1^·cm^−1^ at 260 nm were used to determine the concentration of DNA [56]. The obtained ratio of absorbance of A260/A280 in the DNA sample was 1.97, reflecting the purity of extracted ds. DNA, which was apparently free from proteins. All stock solutions were used within 5 days after preparation and stored at 4 °C until use.

Voltametric assays were performed using a PGZ301 voltammeter running on VoltaMaster 4 V 7.08 software, (Radiometer Analytical SAS, Lyon, France). The concentration of the supporting electrolyte was kept at 0.1 M. Nitrogen gas was bubbled through the solution to avoid any interference from air. Experiments were carried out in a 12 mL three-electrode electrochemical cell containing a platinum disk (Pt) working electrode with a geometric area of 0.013 cm^2^, a platinum wire as counter (auxiliary) electrode and an Hg/Hg_2_Cl_2_ paste-covered wire as reference electrode.

As a complementary method to the voltammetry techniques, electronic spectroscopy titration was performed to study the binding affinity of E_2_ and E_3_ with DNA in buffer phosphate solution (KH_2_PO_4_/K_2_HPO_4_) at pH = 7.2. The absorption spectra of a fixed amount of 2 mg of E_2_ and 6 mg of E_3_ were recorded in the absence and presence of a gradually increasing concentration of DNA stock solution. Absorption spectra measurements were conducted on a UV-Vis spectrometer, (Shimadzu 1800, Japan).

#### 2.3.2. Superoxide Anion Radical Assay

Cyclic voltammetry measurements were performed on a platinum electrode at 100 mV·s^−1^ using a PGZ301 voltammeter running on VoltaMaster 4 V 7.08 software (Radiometer Analytical SAS, France).

In order to obtain kinetic curves and to calculate the IC_50_ values, the O_2_^.-^ radical-scavenging activity was plotted based on Equation (1) against varying compound concentrations [57,58,59]. The antioxidant capacity of E_2_ and E_3_ was expressed as IC_50_. The IC_50_ value was defined as the concentration (mg/mL) of samples that inhibited the formation of O_2_^.-^ radicals by 50%.

(1) $$\%\;radical\;scavenging\;activity=\;\frac{i_{pa0}-i_{pa}}{i_{pa0}}\times 100\;$$

where *i*_*pa*0_ and *i_pa_* are the anodic peak current densities of the superoxide anion radical in the absence and presence of E_2_ and E_3,_ respectively.

#### 2.3.3. DPPH (2,2-Diphenyl-1-picrylhydrazyl) Assay

Spectrophotometric experiments were performed to measure the antioxidant activity of E_2_ and E_3_ samples using DPPH free radical. Experiments were carried out by adding gradually increasing concentrations of E_2_ in DMF (5 mg/mL) or E_3_ (5 mg/mL) to a 2.1 mM DMF solution of DPPH. Absorbance at 524 nm was measured after 30 min of incubation in the dark. Finally, Equation (1) was applied to calculate % inhibition of DPPH. In order to obtain kinetic curves and to calculate the IC_50_ values, DPPH radical-scavenging was plotted against varying compound concentrations. The antioxidant capacity of E_2_ and E_3_ was expressed as IC_50_.

## 3. Results

### 3.1. Biomass Proximate and Ultimate Analyses

Prior to designing a biorefinery approach with the goal of recovering valuable molecules, the feedstock (*Fucus spiralis* macroalgae) was subjected to proximate and ultimate analyses; the chemical composition is listed in Table 1. The *Fucus spiralis* ash content is similar to the values reported in the literature for other algae species: an ash content of 21.0 ± 0.2% dw and 28.0 ± 0.2% dw for *Laminaria digitata* and 25.0 ± 0.2% dw and 26.5 ± 0.7% dw for *Fucus spiralis* [60]. Furthermore, [61] we determined an ash percentage of 31.0 ± 0.1% dw for untreated *Laminaria digitata*, whereas the washed alga generated a value of 7.9 ± 0.1% dw.

The among of reducing sugars detected in *Fucus spiralis* biomass was 16.3%. Lower limits of 9.71 ± 0.03% dw [62] and 10–11% dw [28] were reported, as well as higher concentrations [63]. With respect to the amount of proteins amount, the percentage obtained in the current study for *Fucus spiralis* is close to values registered in the case of *Fucus vesiculosus*, i.e., 12.99% dw [64] or 11% dw [28].

### 3.2. A Biorefinery Approach for Fucus spiralis Macroalgal Biomass Processing

**Figure 1:**
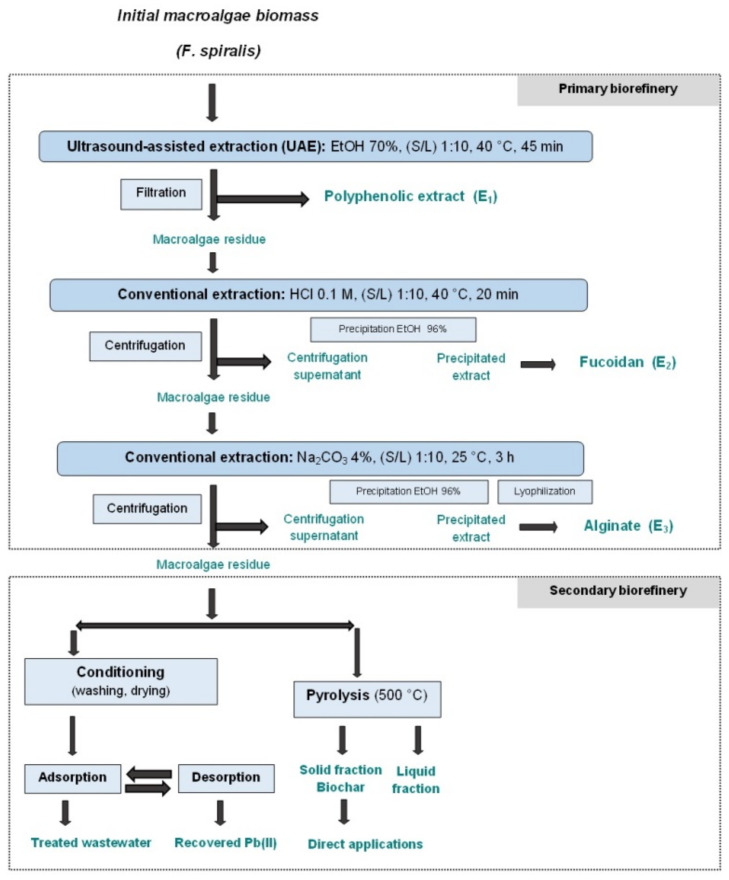
The biorefinery flow sheet designed for *Fucus spiralis* biomass.

Based on the chemical characterization of the feedstock and available studies concerning sequential separation of macroalgal compounds [39,44,48,65], a biorefinery flow sheet for *Fucus spiralis* biomass was designed (Figure 1). All solvents and methods were selected taking into consideration cost effectiveness and circular economy. Thus, a sustainable, efficient and minimal-waste approach was considered. The primary biorefinery step was designed to obtain easily extractible valuable molecules, such as polyphenols and saccharides.

The sequence starts with green light sonication (Figure 1) in order to separate the polyphenols. This step could also act as a pretreatment to make the biomass more suitable for the following extraction steps. Polyphenol separation in the initial biorefinery step is recommended in order to avoid the contamination of further extracted molecules [46,66,67]. Furthermore, *Fucus* is among the brown macroalgae genera with the highest percentage of polyphenolic compounds (1–14% dw) [31]. Usually, polyphenol separation from marine macroalgae is performed using formaldehyde as, as polyphenols have tight chemical bonds with macroalgal polysaccharides [68,69]. However, formaldehyde is known to be a carcinogenic solvent. Therefore, polyphenol extraction was performed with ethanol (70%), considering the positive yields it provides, GRAS recognition (Generally recognized as safe) and low cost [70,71]. Polyphenols were extracted in a batch sonication system using a 1/10 solid/liquid ratio at 40 °C, with an extraction time of 45 min.

For the extraction of fucoidan, hydrochloric acid was selected as solvent, as it has shown satisfactory results in extracting fucoidan in single processing or even in sequential extractions from brown algae [48]. After washing the algal residue in distilled water, alginate is the next compound to be extracted using sodium carbonate [19,44]. A low temperature was chosen in order to avoid the degradation of the targeted metabolites [28,67]. Fucoidan and alginate must be separated sequentially; otherwise, both molecules would be separated in the same extract following the degradation of the algal cell wall [72].

The *Fucus spiralis* solid residue generated as waste after the separation of bioactive molecules was designated as a biosorbent for a cascade of sorption/desorption cycles. Several studies have dealt with the use of macroalgae to uptake pollutants from wastewater due to their effectiveness in the removal of low concentrations and relatively low cost in comparison to conventional adsorbents [73,74,75,76,77]. The literature has proven that brown macroalgae are very good biosorbents and are more effective in the removal of harmful pollutants than red and green algae [78,79]. In most previous studies, including research using *Fucus spiralis* [73], researchers used the entire algal biomass as biosorbent, whereas few studies involved biosorption tests with seaweed waste. Examples include the use of red algae waste generated from agar extraction to adsorb Cu^+2^, Pb^+2^ and Cd^+2^ [80,81,82] and the application of dealginated brown macroalgae waste [83,84]. Residues derived from brown macroalgae *Laminaria digitata* and green macroalgae *Ulva lactuca* were used as biosorbent for Ce^+3^, Pb^+2^, Cu^+2^ and Ni^+2^ [85]. However, only our team has reported satisfactory biosorption results with *Fucus spiralis* waste [75].

Considering all these aspects, the designed biorefinery approach was regarded to be a viable option for the processing of *Fucus spiralis* biomass and was therefore applied in the current study.

### 3.3. Extract Characterization

#### 3.3.1. Yield and Chemical Characterization of Polyphenolic Extract (E_1_)

The *Fucus spiralis* polyphenol extract E_1_ registered a total polyphenolic content (TPC) of 3.4 ± 0.0 mg GAE g^−1^. This value is lower than those obtained in [86] for *Laminaria digitata* (37.66 ± 0.00 mg GAE g^−1^ dry seaweed) and *Laminaria saccharina* (66.75 ± 3.72 mg GAE g^−1^ dry seaweed) collected from Northern Ireland but comparable to those generated for conventional polyphenol separation from *Laminaria japonica* (38.47 ± 0.88 GAE mg/100 g dry seaweed), *Lessonia trabeculate* (49.80 ± 5.68 GAE mg/100 g dry seaweed) and *Ascophyllum nodosum* (51.47 ± 3.28 GAE mg/100 g dry seaweed) [87]. The geographic areas in which the algae originated, as well as the time of collection and pretreatment processes, could explain these differences. The polyphenol extraction yield obtained from *Fucus spiralis* was 14.0% dry seaweed.

#### 3.3.2. Yield and Chemical Characterization of Fucoidan Extract (E_2_)

Fucoidan is an anionic heteropolysaccharide with a linear structure [88]. Fucoidan from *Fucus spiralis* was obtained after the separation of polyphenols with a yield (1.35 ± 0.1% dry seaweed) similar to values reported for brown macroalgae through conventional methods [46] and green methods [36]. The algal polymer extracted from brown species *Sargassum crispifolium* with 80% carbohydrate content by conventional isolation method and using distilled water as solvent generated a yield value of 1.50% dry basis [89]. Using a much higher extraction time (8 h), fucoidan was separated from *Sargassum mcclurei*, *Sargassum polycystum* and *Turbinara ornata* using CaCl_2_ 2%, with yields between 2.10 and 2.75% dried seaweed [90]. However, a separation method that increases the extraction yield is not necessarily favourable for structural integrity, purity and bioactive properties of the extracts [46].

In terms of chemical composition, elemental analysis of the E_2_ extract (Table 2) indicates the presence of sulphur, which pinpoints the fucoidan compound, as well as a high level of carbon (32.1%). Elemental assays in *Fucus serratus* and *Fucus vesiculosus* identified lower carbon values (21–28%) [91]. The amount of sulphur is much lower than that obtained from *Fucus vesiculosus* (9–12%) [34]. The H% content of the obtained fucoidan is similar to the values reported for other brown macroalgae: 4.77% for *Fucus vesiculosus*, 4.78% for *Fucus serratus* [92], 4.64% for *Laminaria japonica* [93] and 4.99% for *Undaria pinnatifida* [94]. Moreover, a 3.47% hydrogen content reported for fucoidan standard [95].

**Figure 2:**
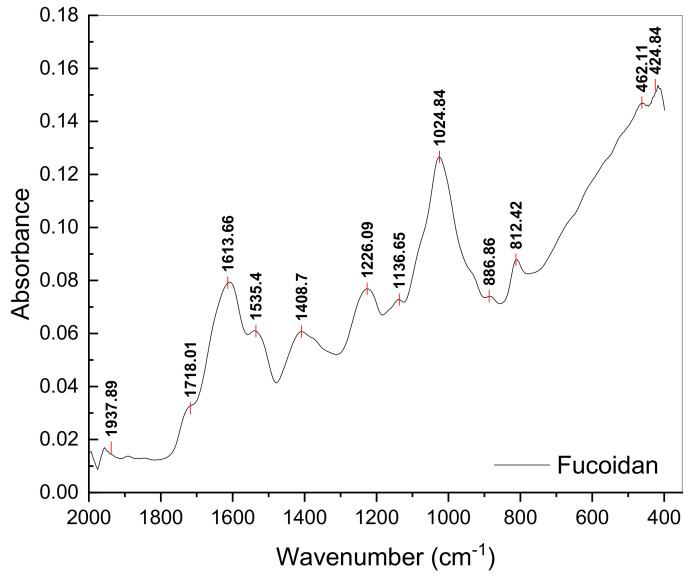
FTIR spectrum of fucoidan extract separated from *Fucus spiralis* biomass.

E_2_ was also subjected to FTIR characterisation (Figure 2). The spectra indicate the presence of carboxyl and carbonyl groups according to the peak registered at 1718 cm^−1^ [96]. Furthermore, an intense peak highlights the presence of the sulphate functional groups (S=O) characteristic of fucoidan at a wavelength of 1226 cm^−1^ [34,97]. Two additional groups, one characteristic of sulphonamides, i.e., N=S=O [98], and one associated with C-O-SO_3_, were observed at 1136 cm^−1^ and at 1024 cm^−1^, respectively. Groups containing sulphur (S) play an important role in determining the bioactive properties of fucoidan [67]. The sulphate functional group of marine macroalgae polysaccharides is associated with the antiviral properties of these compounds [88]. The peak at 1535 cm^−1^ indicates the presence of an N-H protein group in the structure of the amide II. The peak identified at 1408 cm^−1^ was assigned to the C-N group of amide III, whereas that identified at 1613 cm^−1^ was assigned to the C=O functional group of amide I or to the functional groups of uronic acids [98]. Additionally, wavelengths 812 and 886 cm^−1^ were associated with units of mannuronic acid in the structure of alginate [97,99], which indicates that a complete separation of fucoidan could not be achieved.

**Figure 3:**
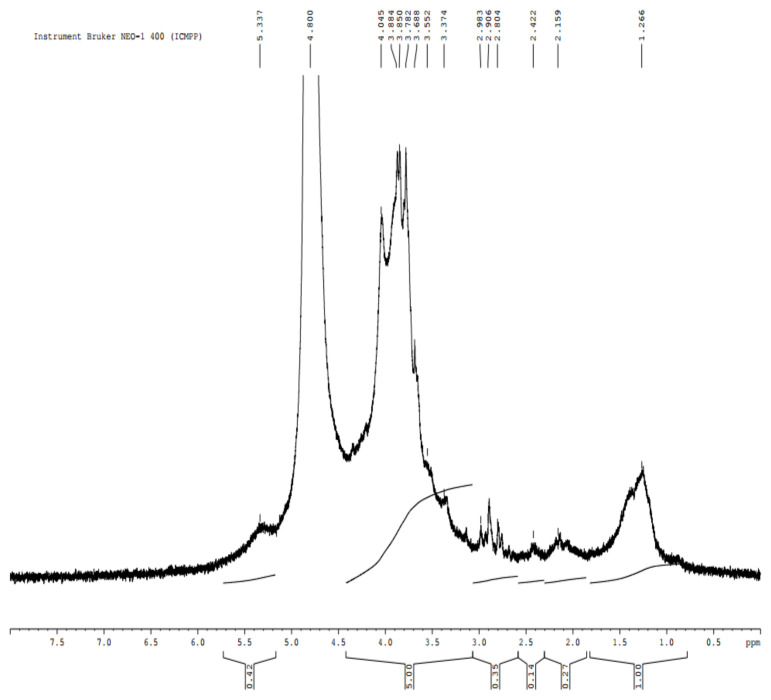
NMR spectrum of fucoidan extract separated from *Fucus spiralis* biomass.

The NMR spectrum (Figure 3) of E_2_ highlights the chemical structures of fucoidan. The peak at 5.337 ppm was assigned to α3-linked 2-mono-*O*-sulfated-L-fucopyranose residues [100]. Another characteristic structure, -α-L-fucose, was identified through the intense signal at 4.8 ppm. The several intense peaks at 3.68-3.88 ppm were attributed to 4-linked 2-mono-*O*-sulfated L-fucopyranose residues [100], whereas the peak at 3.37 ppm was associated with ß-d-xylose [98]. Finally, the signal at 1.26 was assigned to the C6 methyl proton group of L-fucopyranose [100,101].

Only traces of mono- or oligosaccharides in the supernatant and in the residual wash waters obtained from the separation of fucoidan extract were determined. Considering that the acid extraction of fucoidan may also remove any remaining polyphenols [102], the TPC of the supernatant was analysed. The obtained result, 1.68 mg GAE g^−1^, indicates that following fucoidan extraction, a high polyphenolic content is still released equal to more than half of the total polyphenolic content initially separated initially UAE. Some studies have associated the antioxidant activity of fucoidan extracts with the presence of polyphenolic impurities [37].

#### 3.3.3. Yield and Chemical Characterization of Alginate Extract (E_3_)

Alginate is the third targeted bioproduct and was separated with a yield of 47.0 ± 1.0% dry seaweed. This value is significantly higher than the alginate yield obtained from *Fucus vesiculosus* (16.2 ± 3.2% *w/w*) [103]. In a study on *Durvillaea potatorum* an alginate yield of 36.55% *w/w* was reported [104]. Other authors reported a 21.06% *w/w* yield for alginate separated from *Laminaria japonica* using Na_2_CO_3_ 2% for 5 h at 60 °C [41], and a similar value was reported for *Ecklonia radiata* using Na_2_CO_3_ 0.2 M for 120 min at 45 °C [46].

In terms of chemical composition, the elemental analysis of E_3_ (Table 2) shows similar carbon and hydrogen percentage values to those reported in the literature [105]. The amount of sulphur is lower than the alginate fraction from *Sargassum fusiforme* (12.5%) [106]. The presence of sulphur in the composition of the alginate extract can be beneficial for bioactive properties, but it also indicates fucoidan residues.

**Figure 4:**
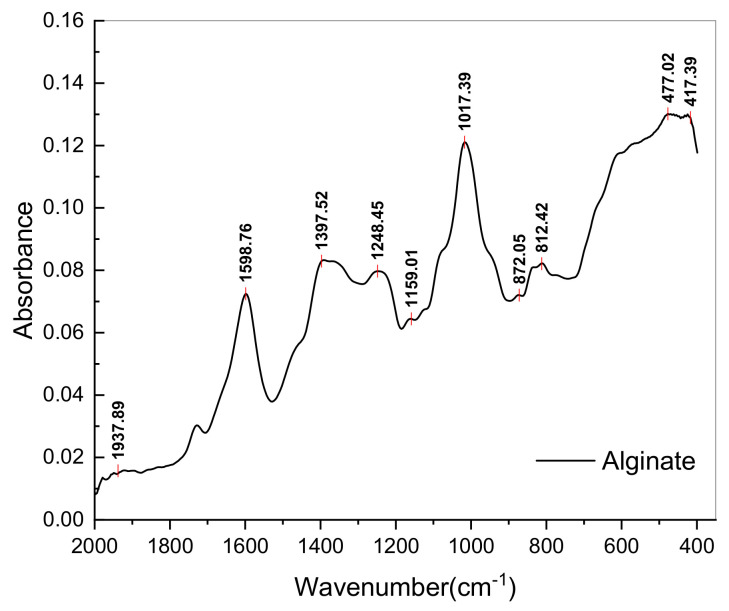
FTIR spectrum of alginate extract separated from *Fucus spiralis* biomass.

Alginate is a marine polysaccharide found in macroalgae, as well as other marine organisms. Its structure comprises homogenous or heterogenous disposition of β-D-mannuronic acid (M) and α-L-guluronic acid (G) [107]. Spectral analysis of E_3_ (Figure 4) identified the presence of guluronic units (G) at 1017 cm^−1^ [96,97,99] and mannuronic units (M) at 872 and 812 cm^−1^ [97,99], confirming the isolation of alginate. The presence of uronic acids is also indicated by the functional groups identified at 1598 and 1397 cm^−1^. Furthermore, sulphate (characteristic of fucoidan) was observed at 1159 and 1248 cm^−1^ [97], suggesting that the extraction was not completely selective.

**Figure 5:**
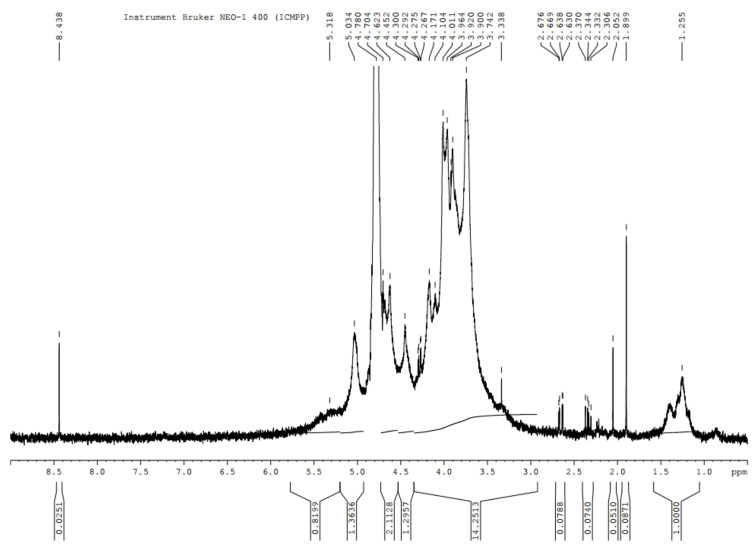
NMR spectrum of alginate extract separated from *Fucus spiralis* biomass.

The ^1^H NMR spectrum of E_3_ representing the uronic acid sequence is depicted in Figure 5. The peak at 5.03 ppm was associated with H^1^ of α-l-guluronic acid [106,108]. The presence of M units in the extract was indicated through an intense peak at 4.62 ppm, which was assigned to β-d-mannuronic acid residues [106,108]. Resonance signals of G units were also identified by peaks at 4.45 and 4.3 ppm [109].

In the supernatant resulting after E_3_ separation, a total polyphenolic content (TPC) of 0.18 mg GAE g^−1^ was determined. The presence of polyphenols is due to the use of ethanol, which facilitates the release of residual aromatic fractions from the cell wall of the macroalgae [19].

### 3.4. Assessment of Targeted Molecule Bioactivity

#### 3.4.1. Electrochemical DNA Binding Study

**Figure 6:**
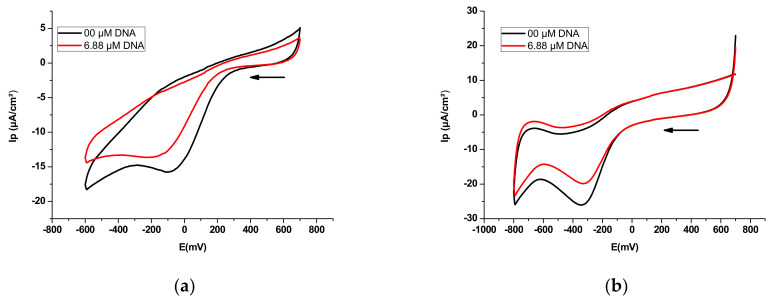
Cyclic voltammograms of polysaccharidic extract in 12 mL of 0.1 M phosphate-buffered solution (KH_2_PO_4_/K_2_HPO_4_, pH = 7.2) recorded at 0.1 V s^−1^ potential sweep rate on a platinum disk electrode at 298 K in the absence and presence of 6.88 µM DNA with supporting electrolyte 0.1 M Bu_4_NBF_4_: (**a**) extract E_2_ (65 mg); (**b**) extract E_3_ (60 mg).

In the absence of DNA, the voltammograms of compounds E_2_ and E_3_ in a phosphate-buffered solution (KH_2_PO_4_/K_2_HPO_4_) show a cathodic peak potential at −94 and −342 mV, respectively. On the other hand, in the presence of a concentration of 6.88 µM of DNA, the peak potential of E_2_ was shifted to a more negative value of −173 mV, with an apparently negative shift of −79 mV, whereas the peak potential of E_3_ was shifted to a more positive value of −335 mV, with an apparently positive shift of +7 mV (Figure 6). These results indicate that E_2_ interacts with DNA via an electrostatic mode [55,56], whereas the obvious positive shifts of peak potentials of E_3_ indicate that the interaction mode may be intercalation between E_3_ and DNA. The voltammograms also show a drop in the cathodic peak current densities, which can be attributed to slow diffusion of the formed DNA-E_2_ and DNA-E_3_ adducts.

The negative shift in the cathodic peak potential that occurred as a result of the addition of DNA to E_2_ was caused by the electrostatic interaction of the anionic drug with the DNA backbone [56,110]; therefore, the obvious negative peak potential shift (cathodic shift) in the CV behaviour of E_2_ with the addition of DNA can be attributed to the electrostatic interaction (H-bonding) between E_2_ and DNA. This negative peak potential shift further indicates that E_2_ residue is easier to reduce in the presence of a negative DNA environment. However, the positive shift observed in the cathodic peak potential caused by the addition of DNA to E_3_ can be explained by the intercalation interaction mode between E_3_ and DNA. This positive shift further indicates that the formed adduct DNA-E_3_ is electrochemically more stable than E_3_.

The binding constant of the interactions of E_2_ and E_3_ with DNA can be calculated based on the decrease in the cathodic peak current density of DNA-E_2_ and DNA-E_3_ adducts relative to free E_2_ and E_3_, respectively, using the following Equation (2) [111]:

(2) $$\log\frac{1}{\left[DNA\right]}=\log K_{b}+log\frac{ip}{ip_{0}-ip}$$

where [*DNA*] is the DNA concentration (M); *K_b_* represents the binding constant (M^−1^); and *ip*_0_ and *ip* denote the cathodic peak current density of the free and DNA-bound compound (μA·cm^−2^), respectively.

**Figure 7:**
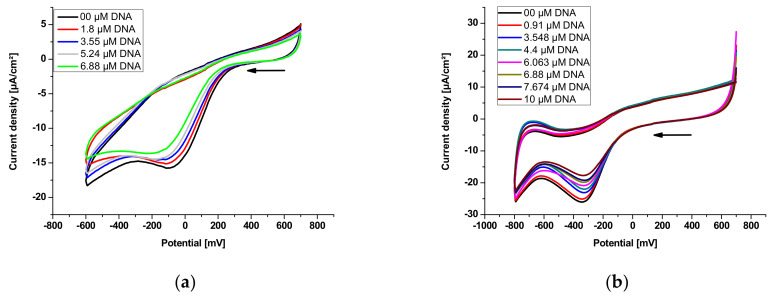
Cyclic voltammograms of polysaccharidic extracts in 12 mL of 0.1 M phosphate-buffered solution (KH_2_PO_4_/K_2_HPO_4_, pH = 7.2) recorded at a 0.1 Vs^−1^ potential sweep rate on a platinum disk electrode at 298 K in the absence and presence of an increasing concentration of DNA with supporting electrolyte 0.1 M Bu_4_NBF_4_: (**a**) E_2_ (65 mg); (**b**) E_3_ (60 mg).

The plot of log 1/[DNA] versus log *ip*/*ip*_0_ – *ip* should afford a straight line with a ‘*y’* intercept equal to the logarithm of binding constant *K_b_*. To obtain the plot log1/[*DNA*] versus log *ip*/*ip*_0_ – *ip*, voltammograms of E_2_ and E_3_ were obtained in the absence and presence of an increasing concentration of DNA (Figure 7).

**Figure 8:**
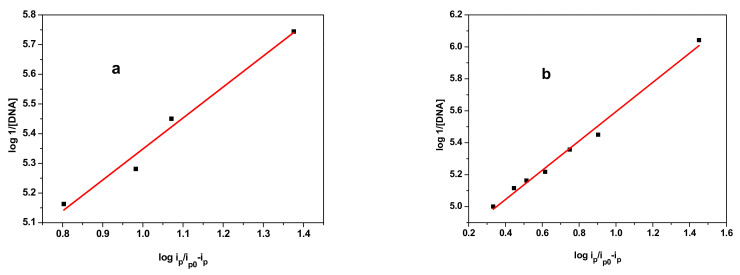
Plots of log (1/1 − (ip/ip0)) versus log 1/[DNA] used to calculate the binding constants of the E_2_ and E_3_ samples with DNA: (**a**) E_2_; (**b**) E_3_.

The plot of log 1/[DNA] versus log ip/ip_0_ – ip is shown in Figure 8, which is a straight line with a ‘*y’* intercept equal to the logarithm of binding constant *K_b_*.

The values of binding constants obtained from the regression of Figure 8 are tabulated in Table 3.

The binding free energy change was calculated using the following Equation (3) [112]:

(3) $$\Delta G=-RT\ln K_{b}$$

where ∆G is the binding free energy in KJ·mol^−1^, R is the gas constant (8.32 J·mol^−1^·K^−1^) and T is the absolute temperature (298 K). Values are also listed in Table 3.

The order of the magnitude of binding site size (s) in terms of the base pair of DNA can provide information about the interaction of small molecules with DNA. The binding site size(s) can be calculated according to the following Equation (4) [113]:

(4) $$\frac{C_{b}}{C_{f}}=K_{b}\left(\frac{free\;base\;pairs}{s}\right)$$

where *s* represents the binding site size in terms of the base pair, *K_b_* is the binding constant, *C_f_* is the concentration of free compound and *C_b_* represents the concentration of DNA-bound compound.

Considering the concentration of DNA in terms of nucleotide phosphate (NP), the concentration of the DNA base pair is expressed as *[DNA]/2,* so Equation (5) can be expressed as:

(5) $$\frac{C_{b}}{C_{f}}=K_{b}\left(\frac{\left[DNA\right]}{2s}\right)$$

The *C_b_*/*C_f_* ratio is equal to (*i*_*p*0_ − *i_p_*)/*i_p_* [114], which are the values of the experimental peak current densities.

The obtained values of binding site size for both samples are summarized in Table 4. The low value of the binding site size of E_2_ further indicates the electrostatic interaction of E_2_ with DNA. In addition, the relatively higher value of the binding site size of E_3_ confirms the intercalation interaction of E_3_ with DNA.

The diffusion coefficient is a parameter that further confirms the interaction of E_2_ or E_3_ with DNA; free E_2_ or E_3_ diffuses more rapidly in solution than their adducts, E_2_-DNA or E_3_-DNA. The diffusion coefficients of the free and DNA-bound forms of E_2_ and E_3_ were determined using the Randles–Sevcik Equation (6) [115].

(6) $$i=2.69\times 10^{5}n^{\frac{3}{2}}ACD^{\frac{1}{2}}v^{\frac{1}{2}}$$

where *n* is the number of electrons per species reaction, *C* is the bulk concentration (mol·cm^−3^) of the electroactive species, *D* is the diffusion coefficient (cm^2^/s) and *v* is the scan rate (V/s).

**Figure 9:**
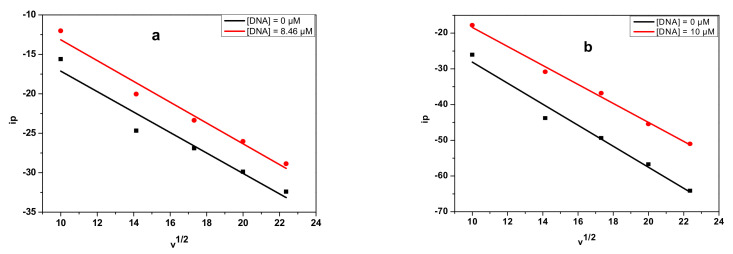
Plots of $\sqrt{v}$ versus *i_p_* used to calculate the coefficient diffusion of the free and E_2_- and E_3_-bound DNA: (**a**) E_2_; (**b**) E_3_.

The plots of $\sqrt{v}$ versus *i_p_* are displayed in Figure 9, whereas the diffusion coefficient values are presented in Table 5. The diffusion coefficients of the DNA-E_2_ and DNA-E_3_ adducts are lower than those for the free E_2_ and E_3_ samples, indicating the formation of a high-molecular-weight complex that diffuses slowly toward the electrode.

#### 3.4.2. Electronic Spectroscopy DNA Binding Study

**Figure 10:**
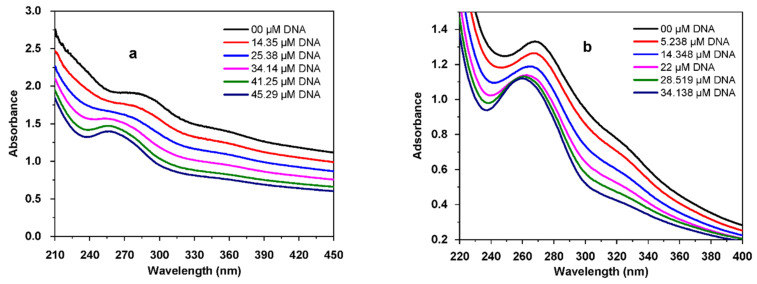
UV-visible absorption spectra of polysaccharidic extracts in the presence of increasing concentrations of DNA in 0.1 M phosphate-buffered solution (KH_2_PO_4_/K_2_HPO_4_) at pH = 7.2 and 298 K: (**a**) 2 mg of E_2_; (**b**) 6 mg of E_3_.

The absorption spectra of E_2_ and E_3_ in the absence and presence of a gradually increasing concentration of DNA stock solution are shown in Figure 10. In the ultraviolet region, E_2_ has one absorption peak at 281 nm (Figure 10a), and E_3_ also displays one peak at 268 nm (Figure 10b).

Upon addition of DNA, a significant hypochromicity was observed without any noticeable shift in the position of the maximum absorption peak, clearly indicating the formation of the adducts E_2_-DNA and E_3_-DNA, with hypochromicity further suggesting the groove-binding property of E_2_ and E_3_ compounds with respect to the double-stranded DNA [116].

Absorbance values were changed by increasing DNA concentration to evaluate the binding constant using the following Equation (7), [117]:

(7) $$\frac{A_{0}}{A-A_{0}}=\frac{\varepsilon_{G}}{\varepsilon_{H-G}-\varepsilon_{G}}+\frac{\varepsilon_{G}}{\varepsilon_{H-G}-\varepsilon_{G}}\frac{1}{K_{b}\left[DNA\right]}$$

where [*DNA*] is the DNA concentration; *K_b_* is the binding constant; *A*_0_ and *A* are the absorbance of E_2_ and E_3_, respectively, in the absence and presence of *DNA*; and *ε_G_* and *ε_H-G_* are their extinction coefficients, respectively.

**Figure 11:**
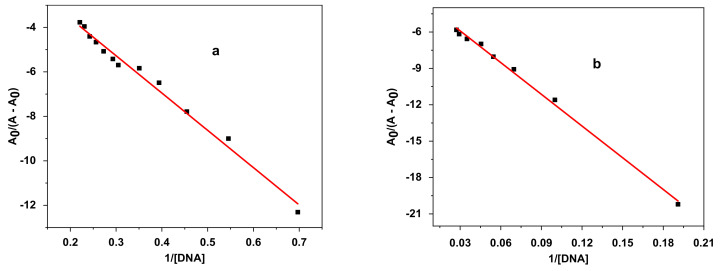
Plots of *A*_0_/(*A* – *A*_0_) versus 1/[*DNA*] used to calculate the binding constants of algal polysaccharidic extracts with DNA: (**a**) E_2_ and (**b**) E_3_.

The binding constant (K_b_) is obtained from the intercept to slope ratio of the plot of A_0_/(A – A_0_) versus 1/[DNA] (Figure 11). The binding free-energy change was calculated using Equation (4). Values for both binding constant and free energy are listed in Table 6.

The values of the binding free energy and binding constant obtained from cyclic voltammetry (Table 3) and electronic spectroscopy (Table 6) are in agreement.

#### 3.4.3. Superoxide Anion Radical Assay

**Figure 12:**
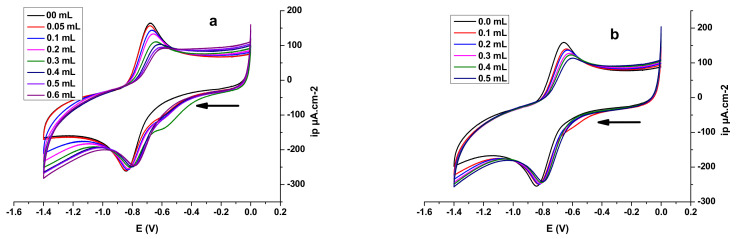
Cyclic voltammograms of oxygen-saturated DMSO/0.1 M Bu_4_NBF_4_ on a Pt electrode in the absence and presence of varying concentrations of algal polysaccharidic extracts at a scan rate 100 mV/s: (**a**) E_2_; (**b**) E_3_.

The one-electron reduction in molecular oxygen generates the superoxide anion radical; this reactive oxygen species causes cell degradation and leads to aging and various diseases [118]. Antioxidants of various natures play a major role in the prevention of these occurrences. Figure 12 shows the decrease in the anodic peak potential of the voltammograms of the radical O_2_^.-^ in the presences of varying concentrations of E_2_ and E_3_.

The equations obtained from the linear calibration graph in the studied concentration range for E_2_, E_3_ and α-tocopherol are summarized in Table 7 (where y represents the value of the anodic peak current density of O_2_^.-^, and x represents the value of the sample concentrations, expressed as mg/mL). All the tests were performed in triplicate, and the graph was plotted according to the averages of three observations. The obtained results indicate that the O_2_^.-^ radical-scavenging activity of E_2_ is slightly higher than that E_3_, and both are higher than that of the standard antioxidant α-tocopherol.

**Figure 13:**
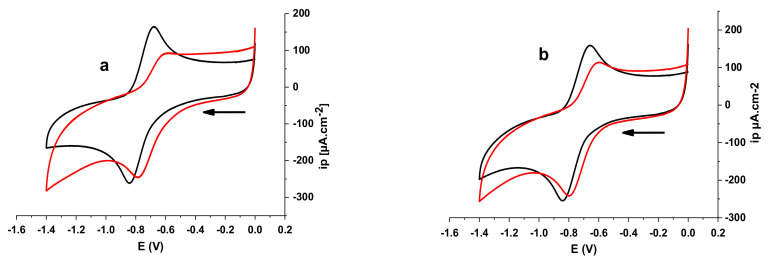
Cyclic voltammograms of O_2_^.-^ on a polished Pt electrode in the absence (black line) and presence of algal polysaccharidic extracts in DMSO solution with supporting electrolyte 0.1 M Bu_4_NBF_4_ at 100 mV·s^−1^: (**a**) 0.6 mL of E_2_; (**b**) 0.5 mL of E_3_.

In the presence of E_2_ and E_3_, the anodic peak potentials of the redox couple O_2_/O_2_^.-^ were shifted to more negative potentials. This shift was associated with a significant decrease in anodic peak current density. The significant drop in anodic peak current density can be assigned to the decrease in O_2_^.-^ concentration due to the formation of slowly diffusing E_2_-O_2_^.-^ and E_3_-O_2_^.-^ complexes. Typical CV behaviour of O_2_^.-^ in DMSO/0.1 M Bu_4_NBF_4_ in the potential window of 0.0 to −1.4 V at a Pt electrode in the presence of E_2_ (a) and E_3_ (b) is shown in Figure 13.

**Figure 14:**
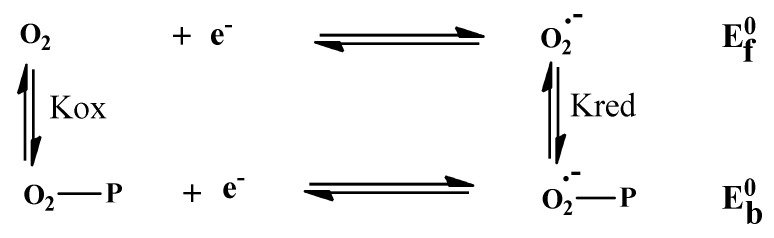
Redox process of the free and E-bound O_2/_O_2_^.-^ redox couple, where E represents E_2_ or E_3_.

The free O_2_/O_2_^.-^ redox couple exhibits one oxidation peak at −0.6764 V and one reduction peak at −0.8411 V. Figure 14 also shows the effect of the addition of E_2_ and E_3_ to a solution of O_2_^.-^ in DMSO/0.1M Bu_4_NBF_4_ on the oxidation peak current density of the O_2_/O_2_^.-^ couple. The decrease in the anodic peak current density caused by the addition of E_2_ or E_3_ can be explained by the reaction of O_2_^.-^ with E_2_ and E_3_ [119]. This decrease can be used to calculate the binding constant, whereas the shift in peak potential values can be exploited to determine the mode of interaction [120,121].

The addition of varying concentrations of E_2_ or E_3_ in DMSO to a solution of DMSO saturated with commercial oxygen provokes a remarkable decrease in the peak current density (Figure 13). The substantial diminution in peak current density can be attributed to the decrease in free O_2_/O_2_^.-^ radical concentration due to the formation of E_2_-O_2_^.-^ or E_3_-O_2_^.-^ product. The gradual decrease in peak current density of the O_2_/O_2_^.-^ redox couple by increasing E_2_ or E_3_ concentrations can be exploited to calculate the binding constant by applying the following Equation (8) [122]:

(8) $$log\left(\frac{1}{C}\right)=log\left(K_{b}\right)+log\left(\frac{i_{p_{a}}}{i_{p_{a0}}-i_{p_{a}}}\right)\;$$

where *i_Pa0_* and *i_Pa_* are the peak currents of the superoxide anion radical in the absence and presence of E_2_ and E_3_, respectively; whereas *C* is the concentration of E_2_ or E_3_. As *C* is not known, this term was replaced by the volume of the added E_2_ or E_3_ (Δ*V*).

The volume of the solution containing O_2_^.-^ is fixed; thus, the addition of volume increments of E_2_ or E_3_ is proportional to the addition of an increased number of moles (i.e., concentration) of the compounds. Binding free energy in this case can be calculated using the following Equation (9) [119]:

(9) $$log\left(\frac{1}{\Delta V}\right)=log\;\left(K_{b}\right)+log\left(\frac{i_{p_{a}}}{i_{p_{a0}}-i_{p_{a}}}\right)$$

In Equation (9), Δ*V* represents the added volume of E_2_ or E_3_ (mL); *K_b_* refers to the binding constant; and *i*_*Pa*0_ and *i_Pa_* are the anodic peak current densities in the absence and presence of E_2_ or E_3_, respectively.

The binding free-energy change was calculated using Equation (4). The values of binding constant and binding free energy are summarized in Table 8.

The addition of 0.6 mL of E_2_ or 0.5 mL of E_3_ has caused a slight shift in peak potential *ΔE^0^* in the negative direction in association with a remarkable decrease in anodic peak current density due to the scavenging activity of the added compounds [123] (Figure 13). The significant drop in anodic peak current density can be assigned to the decrease in O_2_^.-^ radical concentration due to the formation of E_2_-O_2_^.-^ or E_3_-O_2_^.-^ complexes. The peak potential shift of the O_2_/O_2_^.-^ redox couple in the negative direction in the presence of E_2_ or E_3_ indicates that the oxidation of O_2_^.-^ occurs more easily in the presence of E_2_ or E_3_ because its oxidized form, O_2_, is less strongly attached to E_2_ or E_3_ than its reduced form, O_2_^.^. For such a system, where both forms of the O_2/_O_2_^.-^ redox couple interact with E_2_ or E_3_, Figure 14 can be applied [114].

The application of the Nernst relation to the process presented in Figure 14 leads to Equation (10) [124]:

(10) $$\Delta E^{0}=E_{b}^{0}-E_{f}^{0}=E^{0}(O_{2}^{.-}-P)-E^{0}(O_{2}^{.-})=0.059\;log\frac{K_{red}}{K_{ox}}$$

where $E_{f}^{0}$ and $E_{b}^{0}$ are the formal potentials of the O_2_/O_2_^.-^ couple in the free and bound forms, respectively.

The ratio of the binding constants is calculated by replacing ΔE0 from Table 9 in Equation (10).

The obtained ratios of the binding constants indicate that the reaction of E_2_ with O_2_^.-^ is twice as strong as that of E_3_ with O_2_^.-^, which explains the high antioxidant activity of E_2_. Furthermore, values of the ratio of the binding constants indicate that the interaction of the reduced form (O_2_^.-^) of the couple O_2_/O_2_^.-^ with E_2_ or E_3_ is always stronger than that of the oxidized form (O_2_).

#### 3.4.4. DPPH (2,2-Diphenyl-1-picrylhydrazyl) Assay

The antioxidant activity of the macroalgae extracts was also evaluated using DPPH. The equations obtained from the linear calibration graph in the studied concentration range for E_2_ and E_3_ samples and α-tocopherol are summarized in Table 10 (where y represents the value of the absorbance of DPPH, and x represents the value of sample concentrations, expressed as mg/mL). All tests were performed in triplicate, and the graph was plotted with the averages of three observations.

The obtained results presented in Table 10 indicate that the DPPH radical-scavenging activity of E_2_ is slightly higher than that of E_3_, with both approximately equivalent to that of the standard antioxidant α-tocopherol.

**Figure 15:**
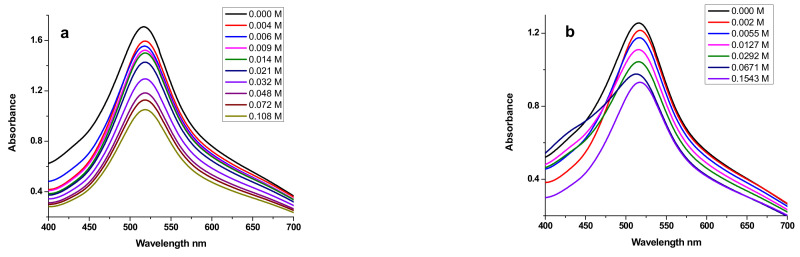
UV-visible absorption spectra of a solution of DPPH (2 mg) in 10 mL acetonitrile in the presence of increasing concentrations of polysaccharidic extracts in acetonitrile at 298 K: (**a**) E_2_; (**b**) E_3_.

The independence of absorbance upon addition of the gradually increasing concentration of E_2_ and E_3_ to a DPPH solution is presented in Figure 15. The figure clearly shows a decrease in absorbance following the addition of the studied compounds.

**Figure 16:**
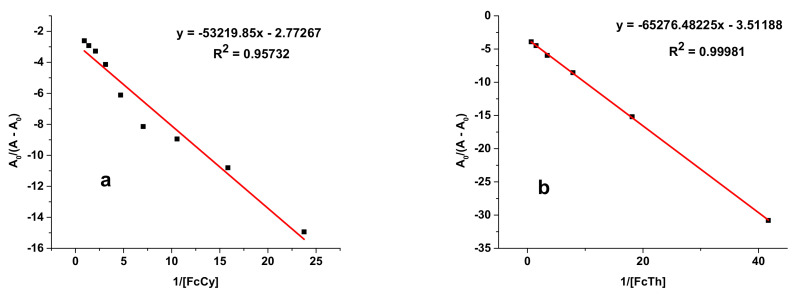
Plots of *A*_0_/(*A* − *A*_0_) versus 1/[*P*] used to calculate the binding constants of algal compounds with DPPH: (**a**) E_2_; (**b**) E_3_.

The change in absorbance values by increasing E_2_ and E_3_ concentration was used to evaluate the intrinsic binding constant by employing Equation (11), [125].

(11) $$\frac{A_{0}}{A-A_{0}}=\frac{\varepsilon_{G}}{\varepsilon_{H-G}-\varepsilon_{G}}+\frac{\varepsilon_{G}}{\varepsilon_{H-G}-\varepsilon_{G}}\frac{1}{K_{b}[P]}$$

where [P] is the E_2_ or E_3_ concentration; K_b_ is the binding constant; A_0_ and A are the absorbance values of DPPH in the absence and presence of E_2_ or E_3_, respectively; and ε_G_ and ε_H-G_ are their extinction coefficients, respectively. The constant K_b_ is obtained from the intercept-to-slope ratio of the plot of A_0_/(A − A_0_) versus 1/[P] (Figure 16).

The binding free-energy change was calculated using Equation (4), and the values are presented in Table 11.

Values of binding free energy listed in the previous table are very close to each other, which may indicate that the studied compounds bind to DPPH in the same manner via their nitrogen atoms.

## 4. Conclusions

In this study, we highlighted a biorefinery approach applied for the first time, at lab scale, to *Fucus spiralis* biomass, with the aim of generating high-value compounds with bioactive properties.

Chemical characterization showed the presence of the targeted algal polysaccharides in the obtained extracts through the identification of S=O (1226 cm^−1^), N=S=O (1136 cm^−1^) and C-O-SO_3_ (1024 cm^−1^) groups characteristic to fucoidan, guluronic, (G) (1017 cm^−1^) and mannuronic units (M) (872 and 812 cm^−1^) specific to the alginate compound.

Cyclic voltammetry and electronic spectroscopy were performed in order to determine the DNA binding affinity and antioxidant activity of E_2_ (fucoidan) and E_3_ (alginate) extracts. A comparison of the binding constant and free-energy values of the generated adducts indicated consistency in the generated results with respect to the interaction of fucoidan and alginate with DNA.

Both polysaccharides demonstrated antioxidant activity, with fucoidan having a slightly higher effect than alginate in both DPPH and O_2_^.-^ radical assays. In DPPH assays, the registered IC_50_ values of E_2_ and E_3_ were 4.49 ± 0.21 and 4.83 ± 0.34 mg/mL, respectively. However, in O_2_^.-^ assays, the obtained IC_50_ values were 0.13 ± 0.04 mg/mL for fucoidan and 0.19 ± 0.06 mg/mL for alginate. Both assays revealed higher antioxidant activity than that of the control antioxidant, α-tocopherol.

## Figures and Tables

**Table 1:** Chemical characteristics of main *Fucus spiralis* biomass.

Property Value	Unit	Value
Proximate analysis		
Moisture content	wt% (dry)	11.5 ± 0.7
Ash content	wt% (dry)	22.5 ± 0.9
Ultimate analysis		
Carbon	wt% (dry)	36.6 ± 0.6
Hydrogen	wt% (dry)	3.9 ± 0.1
Nitrogen	wt% (dry)	1.1 ± 0.1
Sulphur	wt% (dry)	0.5 ± 0.1
Carbohydrate analysis		
Reducing sugars	wt% (dry)	16.3 ± 0.7
	Protein characterization	
Protein content	wt% (dry)	5.7 ± 0.5
Nitrogen-to-protein conversion factor	-	6.25

**Table 2:** Elemental analysis (CHNS) of fucoidan and alginate extracts isolated from *Fucus spiralis* biomass.

Element	Fucoidan	Alginate
C %	32.1 ± 0.4	31.4 ± 0.6
H %	4.0 ± 0.1	5.2 ± 0.3
S %	0.4 ± 0.0	0.3 ± 0.0
N %	ND	3.2 ± 0.2

**Table 3:** Binding constants and binding free-energy values for the interactions of E_2_ and E_3_ with DNA based on CV data presented in Figure 8.

Sample	Equation	R^2^	K (M^−1^)	−ΔG (KJ·mol^−1^)
E_2_	y = 1.0445x + 4.3039	0.972	2.01 × 104	24.57
E_3_	y = 0.9162x + 4.6782	0.989	4.77 × 104	26.71

**Table 4:** Values of binding site size obtained using the plot of Cb/Cf versus (DNA).

Adduct	Equation	R^2^	s
DNA-E_2_	y = 0.02193x − 8.287 × 10^−5^	0.828	0.02
DNA-E_3_	y = 0.0486x − 0.027	0.987	0.12

**Table 5:** Diffusion coefficient values of the free and DNA-bound forms of E_2_ and E_3_.

Compound	Equation	R^2^	D (cm^2^/s)
E_2_	y = −1.29665x − 4.16404	0.938	1.161 × 10^−7^
DNA-E_2_	y = −1.31838 x + 0.02649	0.968	0.721 × 10^−7^
E_3_	y = −2.9437x + 1.3783	0.972	7.086 × 10^−9^
DNA-E_3_	y = −2.6593 x + 8.1874	0.992	5.783 × 10^−9^

**Table 6:** Binding constants and binding free energy values for the interaction of algae polysaccharidic extracts with DNA from the UV data shown in Figure 6: E_2_ and E_3_.

Sample	Equation	R^2^	K (M^−1^)	−ΔG (KJ·mol^−1^)
E_2_	y = −16.76582x – 0.24616	0.986	1.47 × 10^4^	23.79
E_3_	y = −87.1888x – 3.2914	0.998	3.78 × 10^4^	26.13

**Table 7:** IC_50_ values (mg/mL) obtained using O_2_^.-^ radical-scavenging activity.

Sample	Equation	R^2^	IC_50_
E_2_	y = 3.21057x + 0.08397	0.918	0.13 ± 0.04
E_3_	y = 2.3336x + 0.0658	0.984	0.19 ± 0.06
α-tocopherol	y = 15.99x + 1.37	0.950	3.04 ± 0.30

**Table 8:** Binding constant values for E_2_ and E_3_ with O_2_^.-^ from CV data at T = 298 K.

Compound	Equation	R^2^	K (L^−1^)	−ΔG (KJ·mol^−1^)
E_2_-O_2_^.-^	y = 0.96108x + 3.02683	0.978	1.06 × 10^3^	17.28
E_3_-O_2_^.-^	y = 1.28666x + 2.55632	0.891	0.36 × 10^3^	14.59

**Table 9:** Electrochemical data of free and O_2_^.-^-bound forms of E_2_ and E_3_ used to calculate ratio of binding constants.

Compound	Epa (V)	Epc (V)	E0 (V)	$E_{b}^{0}\;-\;E_{f}^{0}\;(V)$	Kred/Kox
E_2_	−0.6764	−0.8411	−0.75875	0.06765	14.01
E_2_-O_2_^.-^	−0.6018	−0.7804	−0.6911		
E_3_	−0.6575	−0.8411	−0.7493	0.05095	7.03
E_3_-O_2_^.-^	−0.6018	−0.7949	−0.69835		

**Table 10:** IC_50_ values (mg/mL) obtained using DPPH radical-scavenging activity.

Sample	Equation	R^2^	IC_50_
E_2_	y = 0.114x − 0.0123	0.997	4.49 ± 0.21
E_3_	y = 0.1051x − 0.0081	0.998	4.83 ± 0.34
α-tocopherol	y =1.5646x + 29.093	0.996	4.81 ± 0.32

**Table 11:** Binding constant and binding free-energy values for E_2_ and E_3_ with DPPH from UV data at 298 K.

Adduct	Equation	R^2^	K (M^−1^)	−ΔG (KJ·mol^−1^)
E_2_-DPPH	y = −53,219.85x − 2.77267	0.957	52.1	9.80
E_3_-DPPH	y = −65,276.482x − 3.5119	0.999	53.8	9.88

## Data Availability

Not applicable.
